# The Role of Telemedicine in Extending and Enhancing Medical Management of the Patient with Chronic Obstructive Pulmonary Disease

**DOI:** 10.3390/medicina57070726

**Published:** 2021-07-18

**Authors:** Claudio F. Donner, Richard ZuWallack, Linda Nici

**Affiliations:** 1 Fondazione Mondo Respiro ONLUS, Via Monsignor Cavigioli, 10, 28021 Borgomanero, Italy; 2 Pulmonary and Critical Care, St Francis Hospital and Medical Center, Hartford, CT 06015, USA; rzuwalla@trinityhealthofne.org; 3 University of CT, Farmington, CT 06030, USA; 4 Pulmonary and Critical Care Section, Providence Veterans Administration Medical Center, Brown University, Providence, RI 02908, USA; linda_nici@brown.edu

**Keywords:** telemedicine, telehealth, integrated care, COPD

## Abstract

Medical management of a chronic obstructive pulmonary disease (COPD) patient must incorporate a broadened and holistic approach to achieve optimal outcomes. This is best achieved with integrated care, which is based on the chronic care model of disease management, proactively addressing the patient’s unique medical, social, psychological, and cognitive needs along the trajectory of the disease. While conceptually appealing, integrated care requires not only a different approach to disease management, but considerably more health care resources. One potential way to reduce this burden of care is telemedicine: technology that allows for the bidirectional transfer of important clinical information between the patient and health care providers across distances. This not only makes medical services more accessible; it may also enhance the efficiency of delivery and quality of care. Telemedicine includes distinct, often overlapping interventions, including telecommunication (enhancing lines of communication), telemonitoring (symptom reporting or the transfer of physiological data to health care providers), physical activity monitoring and feedback to the patient and provider, remote decision support systems (identifying “red flags,” such as the onset of an exacerbation), tele-consultation (directing assessment and care from a distance), tele-education (through web-based educational or self-management platforms), tele-coaching, and tele-rehabilitation (providing educational material, exercise training, or even total pulmonary rehabilitation at a distance when standard, center-based rehabilitation is not feasible). While the above components of telemedicine are conceptually appealing, many have had inconsistent results in scientific trials. Interventions with more consistently favorable results include those potentially modifying physical activity, non-invasive ventilator management, and tele-rehabilitation. More inconsistent results in other telemedicine interventions do not necessarily mean they are ineffective; rather, more data on refining the techniques may be necessary. Until more outcome data are available clinicians should resist being caught up in novel technologies simply because they are new.

## 1. A Brief Review of COPD

Chronic obstructive pulmonary disease (COPD) was conceptualized by the Global Initiative for Chronic Obstructive Lung Disease (GOLD) in 2021 [1] as a common, preventable, and treatable disease characterized by persistent symptoms and airflow limitation. It goes on to say that the airflow limitation can be due to combinations and varying degrees of alveolar abnormalities (i.e., emphysema) and small airway obstruction. Both of these processes are underpinned by chronic inflammation. However, the authors of GOLD point out that the terms “emphysema” and “chronic bronchitis” are not used in the current definition of COPD, because emphysema is a pathological term (distension and destruction of alveolar units) that is often misused clinically, and chronic bronchitis is a clinical term (cough and sputum production for at least 3 months over 2 consecutive years), is not particularly common, and can be present without airflow limitation. Rather, the GOLD contributors emphasize the need for the spirometric demonstration of airway obstruction in the appropriate clinical setting. Using an analogy for a systemic hypertension diagnosis (perhaps a bit outdated) the “140/90” for COPD is a forced expiratory volume in one second (FEV1)—a forced vital capacity FVC ratio (FEV1/FVC) < 0.70 on post-bronchodilator spirometry. Spirometric severity is then determined using the percent predicted FEV1 based on comparison with normative reference values. The most common and frequently overriding symptom in COPD is exertional dyspnea.

As stated above, post-bronchodilator spirometry is necessary to confirm the diagnosis of COPD (in the appropriate clinical setting) and determine the degree of airway obstruction. This information, although necessary, is not sufficient to capture the full impact of this disease on the individual. With respect to respiratory physiology, dynamic hyperinflation of the lung is at least as prominent a factor in exertional dyspnea and exercise limitation as the degree of airflow limitation [2,3]. Furthermore, COPD is best considered a disease that has additional effects apart from just the respiratory system, with frequent associated systemic and co-morbid conditions [4,5]. Some of the systemic consequences or co-morbid associations contribute significantly to dyspnea; these include reductions in ambulatory muscle mass or oxidative enzymes, associated cardiovascular disease, fear of dyspnea-producing activities, and improper pacing techniques, to name a few. [6,7,8] Although exercise training, as delivered in a comprehensive pulmonary rehabilitation program, does not affect airflow limitation, it nevertheless results in reductions in dyspnea—presumably through mitigating the effects of the above-mentioned conditions.

Systemic effects of COPD, in addition to aggravating exertional dyspnea, lead to detrimental outcomes in other ways. For example, it has been known since 1996 that a low timed walk distance, arguably a measure of the overall “protoplasm” of a patient, was a stronger predictor of mortality than FEV1 in COPD patients completing pulmonary rehabilitation [9]. This was exemplified by the development of a multi-component staging system, BODE (body mass index, airway obstruction (FEV1), dyspnea (Medical Research Council rating), and exercise capacity (six minute walk distance)) which was a stronger predictor of survival in COPD than any component alone [10]. Other studies showed that dyspnea level, leg or arm muscle depletion, depression, and co-existing cardiac disease also predict all-cause mortality in COPD [11,12,13,14,15,16].

Finally, COPD can be characterized not simply as a progressive disease that leads eventually to an increasing symptom burden, but one that is commonly punctuated by exacerbations. A universally accepted definition of COPD exacerbation unfortunately does not exist, probably reflecting the heterogeneity of COPD phenotypes in general and the exacerbation symptoms in particular. Two basic categories of exacerbation definitions exist: symptom-based and health care-utilization-based. The former refers to the increased symptom burden of the exacerbation, while the latter (often used in pharmaceutical research studies) refers to associated increases in health care resources, such as bronchodilators, antibiotics, or health care visits. Neither is perfect. One relatively new suggested definition is increased sputum volume or sputum purulence that is sometimes, but not necessarily, accompanied by an increased cough, assuming other etiologies (such as heart failure) are ruled out [17]. Regardless of its definition, exacerbation is a major driver of increased symptom burden, reduced health-related quality of life, reduced functional status, and increased health care utilization and mortality risk. There is some evidence that its early recognition—and resulting early treatment—may reduce its severity. Because of this, collaborative self-management strategies, resulting in early detection by the patient and establishing effective lines of communication with the health care provider, are essential components of an effective COPD management strategy.

The “take home message” from the above very brief review of COPD is that, for optimal clinical management, focusing simply on airway obstruction or even on the respiratory disease is too short-sighted. Instead, a broadened, more holistic approach is necessary to achieve optimal outcomes. Enhanced care of this type may benefit from the integration of care, perhaps augmented by newer technologies, such as telemedicine. This will be the main focus of the remainder of this review.

## 2. Integrated Care and the Chronic Care Model of Disease Management

A workshop on the integrated care of COPD hosted by the American Thoracic Society in 2012 defined this intervention as: “The continuum of patient centered services organized as a care delivery value chain for patients with chronic conditions with the goal of achieving the optimal daily functioning and health status for the individual patient and to achieve and maintain the individual’s independence and functioning in the community” [18]. This approach moves away from the acute care to the chronic care model of disease management. Described in 2002; the latter includes management support, clinical information systems, delivery system redesign, decision support in the form of clinical guidelines, health care organization, and the utilization of community resources [19,20]. Integrated care addresses proactively the medical, social, psychological, and cognitive needs of the COPD patient across the trajectory of the disease course [21]. Integrated care and chronic disease management are virtually synonymous concepts, although the former may stress the coordination of care somewhat more than the latter [21].

COPD management strategies that are similar to integrated care (although less broad in scope), include collaborative self-management [22], care coordination [23], and patient-centered medical home [24]. Self-management is a key component of integrated care, aimed at enhancing patient self-efficacy through education aimed at topics such as smoking cessation, the early recognition and prompt treatment of respiratory exacerbation, nutritional support, and stress management. Care coordination, which is a necessary (but not sufficient) intervention to realize optimal outcomes in COPD, must be blended with self-management for optimal outcome. Patient-centered medical home emphasizes the role of the primary care provider in care and, as such, undervalues the role of the specialist physician in clinical management. The above interventions are time-consuming to provide to the COPD patient under the present system. Pulmonary rehabilitation, with components of exercise training and collaborative self-management strategies [25], allow for more time (often weeks) of ongoing health care provider input and coordination of care. Because of this, promotes the integrated services needed to optimize care and thereby enhance outcomes.

## 3. Telemedicine

A reasonable definition of telemedicine, published in 1997, is “the use of electronic and communications technologies to provide and support health care when distance separates the participants” [26]. More recently (2010) the World Health Organization defined telemedicine as “the delivery of health care services, where distance is a critical factor, by all health care professionals using information and communication technologies for the exchange of valid information for diagnosis, treatment and prevention of disease and injuries, research and evaluation, and for the continuing education of health care providers, all in the interests of advancing the health of individuals and their communities” [27]. “Distance” is common to both definitions, as technology has the capacity to create or enhance ongoing bi-directional communication over two or more remote locations.

This type of intervention is supportive to integrated care: alone, it generally is not sufficient to achieve optimal outcomes. Telemedicine is especially useful when provider care or access to providers is limited (such in rural areas), or when ongoing monitoring of unstable conditions and enhanced patient–health care provider communication are necessary. Prime examples of the latter include the post-discharge period following COPD exacerbation and managing non-invasive ventilation adherence and therapy in the patient with chronic respiratory failure [28].

Telemedicine encompasses several distinct, often overlapping interventions, including: (1) telecommunication (enhancing lines of communication between patients, patients’ families and support individuals, and health care providers); (2) telemonitoring (ongoing symptoms or the physiological transfer of data to the health care providers, including symptom ratings, peripheral oxygen saturation, vital signs, weights); (3) physical activity monitoring and feedback to the patient and provider; (4) remote decision support systems (identifying “red flags”, such as the onset of an exacerbation); (5) tele-consultation (directing assessment and care from a distance); (6) tele-education (using such modalities as web-based educational or self-management platforms); (7) tele-coaching; (8) remote diagnosis; and (9) tele-rehabilitation (providing supplemental educational material, exercise training, or even total pulmonary rehabilitation at a distance when standard, center-based rehabilitation is not feasible) [29,30].

Telemedicine should permit clinicians to reach a wider audience, especially those who otherwise might not have received medical intervention. An example of its potential usefulness is the dramatic increase in global telemedicine usage during the 2020–2021 COVID-19 pandemic [31,32], spawned by the need for continuing medical therapy when direct, patient–caregiver interactions were fraught with risk to medically vulnerable patients. The feasibility, benefits, and overall patient satisfaction of telemedicine encounters became clear over those months. Furthermore, its implementation helped relieve some of the burden imposed on health care systems pushed to their limits by required in-person encounters of high acuity. Recognizing this, some health care systems put telemedicine on an equal or near-equal footing as in-person visits for financial reimbursement [33]. 

### 3.1. Telemonitoring

As stated above, telemonitoring as a subset of telemedicine provides for ongoing, real-time information transfer over distance. It generally involves the electronic transfer of symptoms and clinical status from the patient to the health care providers. Information transfer in the other direction (i.e., from the health care provider to the patient) is not strictly monitoring, but part of the larger concept of telemedicine. Since information transfer without agency does not make sense, the following discussion will encompass both modalities. For COPD patients, information transfer from the patient to the health care provider can range from symptom reporting (dyspnea, cough, changes in sputum characteristics, etc.) to clinical data transmittal e.g., vital signs, oxygen saturation by pulse oximetry, body weight. Data transfer can involve single variables or combinations in the form of dashboards, which can provide a “snapshot” of the clinical and health status of the patient. The general idea is that negative changes in these variables can signify clinical deterioration in health - not just from the respiratory disease but also from its co-morbid conditions, such as heart failure decompensation. Early recognition of these changes will presumably lead to early treatment and improved outcomes.

A recent systematic review of studies testing telemonitoring in COPD over the preceding six years [34] found that improved outcomes or satisfaction were reported in 13 out of 29 studies, and reduced need for in-person (primary care or emergency department) visits in 9 of 16 studies. Qualitative assessments of the process from the investigators as gleaned by the reviewers found the following top facilitators: (1) improved outcomes and patient satisfaction (in the positive studies), (2) reduced need for in-person visits, (3) enhanced disease management, and (4) improved relationships between patients and health care providers. Frequent barriers cited included: (1) poor or absent quality outcomes (in the negative studies), (2) low-quality data, (3) an increased workload for providers, and (4) increased cost. Thus, in general, the results from telemonitoring interventions have had mixed results—no doubt reflecting the modes of data transfer and subsequent health care interventions. As is frequently the case, “the devil is in the details” when trying to determine the optimal approach to an intervention such as telemonitoring based on the analysis of varied and complex interventions in the current medical literature.

### 3.2. Promoting Education and Self-Management Support

Educational intervention for the individual with COPD can have different, although overlapping, approaches: (1) increasing the patient’s knowledge of the disease, its treatments, and common co-morbid conditions through didactic approaches; and (2) promoting adaptive self-sufficiency through collaborative self-management training. The latter has goals including adopting a healthy lifestyle (including regular physical activity and exercise), promoting adherence to the varied pharmacologic and non-pharmacologic treatments often necessary to optimize outcomes for COPD, and developing better lines of communication between patients and health care providers.

Education may be enhanced through the use of online classrooms or programs specifically designed for this purpose for patients with COPD. While real-time education is possible with telemedicine, recording of the educational sessions and subsequent play-back by the patient at his/her convenience (with the potential for repeating the session, if needed) is an attractive option. In addition, newer technologies may provide resources to augment self-management goals, especially in situations where access to direct educational input is not feasible or available, such as in health care systems with limited educational resources, in rural areas, or for individuals who are homebound or have limited mobility options.

An example of a web-based platform that provides education (among other interventions such as exercise training) is SPACE for COPD, [35] described as a web-based pulmonary rehabilitation program that includes education and self-management training. Another example of an online, proprietary, monthly-fee-based initiative for COPD is LIFT (https://liftclass.com/pulmonary, accessed 16 July 2021), which provides educational support in the form of multiple available sessions as well as home-based exercise training. This program could be accessed by a patient using a smartphone, tablet, or computer. The efficacy of initiatives such as this remains to be proven in randomized, clinical trials, but the convenience of home-based sessions available to patients any time they wish seems desirable.

One form of self-management education is promoting adherence to regular medication use, such as inhaled corticosteroids (often combined with long-acting beta-agonist bronchodilators) as maintenance therapy for those with asthma–COPD overlap syndrome. Electronic devices are available that can record the number (along with dates and times) of inhalations. While not strictly telemedicine by definition (which typically includes treatment or information transfer at a distance), direct feedback from the clinician to the patient of this information may prove useful in initiating discussion on ways of improving adherence, when necessary. [36]

A Cochrane review published in 2017 [37] compared telemedicine-supported self-management to other approaches to self-management education. This review included randomized, controlled trials that used technologies such as computers, smartphones, etc., to promote self-management behavior change in COPD. Three studies totaling 557 COPD patients were included in the analysis: 319 received technology-supported self-management and 238 received face-to-face verbal, written, or digital information and education about self-management. Compared to conventional approaches, technology-supported self-management resulted in greater improvement in health-related quality of life and physical activity out to 6 months (but not out to 12 months). There was no significant difference between the groups in hospitalizations or exacerbations.

Despite its proven benefit for prolonging life in hypoxemic COPD patients and coverage by third party payers who meet eligibility criteria, adherence to the regular use of supplemental oxygen therapy is sub-optimal, ranging from 45% to 70% [38]. The remarkable success of telemedicine in a large, randomized controlled trial that provided ongoing education and direct feedback to obstructive sleep apnea patients to substantially increase adherence to continuous positive airway pressure (CPAP) therapy [39] should provide an impetus to employ similar technological interventions to augment supplemental oxygen adherence for COPD patients. Considering the success of telemedicine on adherence to CPAP in sleep apnea, one editorialist suggested developing sensors to record ongoing oxygen use and transmit this information to health care providers [40] (and patients as well).

### 3.3. Early Recognition and Management of the COPD Exacerbation

As stated earlier, COPD exacerbation is associated not only with increased symptoms and health status deterioration, but also with increases in health care utilization and mortality risk. Managing COPD exacerbation includes: (1) its early recognition and prompt treatment through the promotion of collaborative self-management, and (2) managing a complex patient, often with severe disease and co-morbidities, during a clinically severe exacerbation. Telemedicine approaches such as telemonitoring and the promotion of collaborative self-management skills may help in self-management through providing feedback to health care providers on changes in clinical status, such as increased cough, dyspnea, fatigue, etc. Information transfer in the other direction—from provider to the patient—may then initiate the augmentation of treatment, when necessary. This may be particularly useful in a COPD patient who is a frequent exacerbator. While multiple factors predict frequent exacerbations in COPD, a history of previous exacerbations ranks high on the list, so special attention to these at-risk individuals using telemedicine may be in order.

Under some health care systems, COPD patients are discharged from hospital on average in about 6 days despite the fact that clinical exacerbation typically lasts about 12 days, with an interquartile range 6–26 days [41]. This necessitates extending the care of the destabilized COPD patient into the home. The discrepancy between the prolonged exacerbation and shortened inpatient care provides one reason behind the approximately 20% 30-day any-cause hospitalization rate for COPD in the United States [42]. It seems plausible that those interventions that prolong the level of care over the days or weeks of this vulnerable period would benefit the patient. The use of telemedicine in the post-discharge period may prove useful in this regard, through facilitating symptom and vital sign reporting to the health care provider and providing ongoing feedback from the patient to the provider and vice versa. This monitoring would include not only respiratory variables such as cough and dyspnea scores and oxygen saturation measurements by pulse oximetry, but also monitoring for co-morbid exacerbations such as decompensation of heart failure but methods such as daily weight monitoring. Additionally, since exacerbations tend to cluster temporally [43] ongoing monitoring may lessen the risk of another clinical deterioration through early detection and prompt treatment.

Despite the rationale for its use, randomized trials of telemedicine evaluating its effectiveness for the COPD exacerbation have had disappointing results. This is underscored by a 2020 systematic review and meta-analysis [44] in which the pooled results from six trials showed a positive trend (relative risk = 0.67, *p* = 0.29) but the outcomes were not statistically different. However, when only those studies that went for longer than 6 months were included or when pulmonary function data were monitored, were significant reductions observed.

While trials of telemedicine have had mixed results in systematic reviews, a randomized, 12-month, multicenter, controlled clinical trial of telemedicine (versus usual care) (PROMETE II) in patients with severe COPD [45] is briefly reviewed here as an example of a well-conducted study that yielded disappointing results. Two hundred and twenty-nine COPD patients were randomized to telehealth or routine clinical practice. Inclusion criteria included severe airway obstruction, supplemental oxygen requirements, and frequent exacerbations. The intervention consisted of a home visit by a nurse, the installation of home monitoring equipment (pulse oximeter, blood pressure gauge, spirometer, and a respiratory rate and oxygen therapy compliance monitor connected to the oxygen source), and the training of the patient and caregivers on this equipment. Blood pressure, oxygen saturation, heart rate, and spirometry were measured by the patient, while respiratory rate and oxygen use adherence were transmitted. Changes beyond pre-set thresholds triggered alerts, and if data were not transmitted by the patient for two days, a telephone call was made to that patient. Disappointingly, despite what appears to be a well-designed and reasonably powered study, the frequency of exacerbations requiring hospitalizations or emergency department visits (the primary outcome) was not significantly different between the two groups. Hospital days and ICU days tended to be lower in the telehealth group, but the differences were not significant. There was no difference in mortality or cost analysis. These negative results, while only representing one study, must temper initial enthusiasm with telemedicine in mitigating the effects of COPD exacerbations.

### 3.4. Promoting Physical Activity

Physical inactivity is common in individuals with COPD and [46,47] relates somewhat to markers of disease severity [46]. Activity assessment in COPD is important, since lower levels of self-reported physical activity are associated with increased health care utilization and increased mortality risk [48,49,50,51]. Despite its prevalence in COPD and association with poor outcome, a 2016 systematic review of 60 interventional studies aimed at increasing activity, including counseling, pulmonary rehabilitation, and bronchodilators, reported modest success in this important therapeutic area [52]. The sustained behavior change needed to produce sustained increases in physical activity is not easily achieved. An accompanying editorial concluded that coaching interventions and longer-lasting pulmonary rehabilitation programs have the greatest potential to favorably modify physical activity [53].

Since self-monitoring of daily activities using an activity monitor, accompanied by feedback and goal setting, is an important component of activity counseling and coaching, the use of telemedicine technology may assist in this regard. Indeed, a 2014 systematic review of nine studies totaling 982 patients did report a beneficial effect of tele-health care on physical activity levels, with an increase of about one hour of physical activity behavior over comparators (usual care, exercise training, and education) [54]. The interventions cited in this review included telephone calls and websites, typically combined with education and/or exercise training.

### 3.5. As an Adjunct or Alternative to Pulmonary Rehabilitation

The 2013 American Thoracic Society–European Respiratory Society definition of pulmonary rehabilitation is “… a comprehensive intervention based on a thorough patient assessment followed by patient-tailored therapies that include, but are not limited to, exercise training, education, and behavior change, designed to improve the physical and psychological condition of people with chronic respiratory disease and to promote the long-term adherence to health-enhancing behaviors” [25]. Although exercise training is a required component of pulmonary rehabilitation, as a sole intervention it is considered not sufficient for optimal outcome. Compared to usual care, pulmonary rehabilitation leads to substantial improvements in dyspnea, exercise capacity, and health status [25]. Furthermore, when provided following hospitalizations for COPD exacerbation, it has pronounced benefit in reducing subsequent hospitalization and mortality risk [55], and referral leads to projected favorable benefit–cost analyses, both from a systematic review [56], and across different health care settings in the United Kingdom [57].

While standard, center-based pulmonary rehabilitation has proven to be highly effective across multiple outcome areas, it remains grossly under-utilized across most health care systems [58]. Barriers to its under-utilization include insufficient funding, lack of availability, and under-awareness of the availability or benefits of the intervention by patients, health care providers, and governmental or insurance payers [58]. Utilizing telemedicine technologies to provide or enhance pulmonary rehabilitation (tele-rehabilitation) may allow for greater health care access and service delivery options, especially for those geographically or socially isolated, for those with time conflicts such as regular employment, or for those with disease or co-morbid severity that would preclude regular visits to a pulmonary rehabilitation center. Ref. [59] Indeed, studies utilizing questionnaires or interviews have demonstrated that the utilization of technology to support pulmonary rehabilitation met with high levels of patient satisfaction, possibly explaining the higher levels of adherence associated with its use [60]—at least out to two years [61].

A 2021 Cochrane review of tele-rehabilitation [62] included 15 studies totaling 1904 patients, 99% of whom had COPD. Exercise training was required for inclusion in the review, with at least 50% of the intervention given via tele-rehabilitation. Two major comparisons were made: (1) tele-rehabilitation versus standard pulmonary rehabilitation, and (2) tele-rehabilitation versus usual care. For the former comparison, the reviewers found little or no difference in the following post-rehabilitation outcomes: six-minute walk distance, health-related quality of life, or breathlessness. Patients randomized to tele-rehabilitation were more likely to complete the program than those randomized to standard rehabilitation: 93% versus 70%. For the latter comparison (tele-rehabilitation versus standard care) increases from the intervention were observed in the six-minute walk distance, with a mean difference of 22 m, although that is below the 35 m threshold for what is considered a clinically significant response. However, when tele-rehabilitation is used as maintenance therapy, the mean difference of 78 m surpassed this threshold. Heath care utilization data were not presented. No adverse events over and above standard rehabilitation were noted, and no significant data on long-term effectiveness was available. The authors correctly stated that, while providing remote pulmonary rehabilitation has potential benefits (cited above), the lack of in-person supervision and peer support that characterizes center-based interventions may adversely affect rehabilitation outcomes.

In the era of required social distancing because of the COVID-19 pandemic, almost all of the center-based pulmonary rehabilitation programs temporarily shut down, and at the time of this paper they have still only re-opened to a limited degree. This has led to the use of telemedicine technology to provide elements of pulmonary rehabilitation to those who need it, yet who—because of their age, disease severity, and frequent co-morbid conditions—would be at risk in the crowded settings of traditional pulmonary rehabilitation [63]. How this will play out once the pandemic subsides remains to be determined.

A major tenet of comprehensive pulmonary rehabilitation is that patients need to continue health-promoting behaviors gained after the formal program is over, and the use of tele-rehabilitation to promote the maintenance of gains remains a fertile area for scientific exploration. One pilot study (10 patients with COPD) published in 2017 did test the utility of tele-rehabilitation promotion of ongoing exercise after the center-based program was over. Tele-rehabilitation maintenance included home exercise, telemonitoring, and webpage-based self-management, as well as weekly videoconferencing sessions. Gains across multiple outcome areas were maintained out to two years in this small study [64]. A considerably larger randomized controlled trial [65], also published in 2017, compared home-based maintenance tele-rehabilitation (*n* = 47) and center-based maintenance rehabilitation (*n* = 50) out to 12 months after the formal intervention. The study also included outcome assessment at 12 months of usual care treatment (*n* = 50) without initial pulmonary rehabilitaton. Both forms of maintenance resulted in a lower risk for COPD exacerbation as well as hospitalization for exacerbation compared to usual care. Functional exercise capacity, health status and physical activity were also maintained, in a fashion superior to usual care. Adherence to the tele-rehabilitation maintenance was over 93%. The authors concluded that the excess cost of home-based tele-rehabilitation maintenance (1800 EUR) was overshadowed by the estimated reduction in costs by reducing health care related to fewer exacerbations (2908 EUR).

Despite the potential benefits of telemedicine as a tool for better delivering health care for COPD patients and the above study of maintenance tele-rehabilitation as a maintenance intervention, because of its generally higher costs and variable outcomes, its cost–benefit remains uncertain [66]. Because of this, further studies are necessary to draw firm conclusions [67].

### 3.6. In Managing the Patient with Chronic Respiratory Failure

Noninvasive ventilation (NIV) is a standard of care in the management of individuals with severe respiratory disease associated with COPD [68], especially for those with acute on chronic hypercarbic respiratory failure. This is supported by a 2017 Cochrane review which demonstrated a significantly reduced need for endotracheal intubation and reduced risk for subsequent mortality [69]. Benefits from NIV for stable COPD patients with chronic hypercarbic respiratory failure are also demonstrated in a 2017 review of seven studies involving 810 patients [70], with positive outcomes of decreased PaCO2 and reduced exacerbation frequency, and improvements in lung function, respiratory muscle function, and exercise capacity, along with decreased mortality.

Despite its demonstrated benefits, because of the severity and complexity of patients’ conditions and the required intensity and time requirements of NIV, health care systems remain challenged in meeting the high costs of these services. This often results in an increased burden for patients, caregivers, and families, resulting from the transition from higher-cost hospitals to lower-cost home care or institutions with less professional input [71]. The use of telemedicine may provide some assistance with this transition, although it has been reported that a sizeable percentage of patients requiring home mechanical ventilation may be resistant to remote monitoring [72], and a survey of home mechanical ventilation patients residing in 11 European countries revealed that approximately one-half reported they would be confident using a telemonitoring system as part of their home mechanical ventilation program [72,73].

In addition to assisting in the ongoing clinical management of chronic respiratory failure treated with NIV, telemedicine may be a useful tool to initiate NIV in the home setting rather than the more costly hospital setting. This potential use was explored in a randomized controlled trial of 67 COPD patients with chronic hypercapnic respiratory failure, who were randomized to either initiation of NIV in the hospital or while at home using telemedicine [74]. Physiological measurements sent to the health care providers for the latter include the transmission of ventilator data and transcutaneous PO2. v Changes in ventilator settings could be performed remotely. A visit by a specialist nurse was made on the initiation day to go over the equipment, explain all procedures, and to help the patient become familiar with NIV. The primary outcome, reduction in PaCO2, was achieved compared to baseline in both groups at six months follow-up, and the magnitude of this response met criteria for non-inferiority. Likewise, health status improved in both, with non-inferiority in the telemedicine group. It was of importance that home NIV initiation using telemedicine was significantly cheaper than initiation in the hospital: (median) €3768 versus €8537. These promising results suggest a potential wider role for telemedicine assisted NIV in the home setting.

Taking into consideration the potential for telemedicine as an adjunct to the use of NIV, a European Respiratory Society Task Force created an official statement, *Tele-monitoring of ventilator-dependent patients* [75]. The authors acknowledged that the high cost associated with hospital management of ventilator patients leads to transfer of patient management to the home setting, thereby increasing the burden of care on families. The review of literature in this document pointed out the potential utility of remote health monitoring systems for ventilator patients, and recommended that national and European Union governments develop guidelines for the ethical, legal, regulatory, technical, and administrative standards for this form of telemedicine. Potential legal issues will need to be addressed, and research to evaluate cost-effectiveness will be necessary.

## 4. Conclusions

The principles of optimal medical care of a complex COPD patient, blending pharmacologic and non-pharmacologic therapies, remain roughly the same whether or not telemedicine technologies are employed. While clinicians should resist being caught up in novel technologies, telemedicine holds promise, and should be considered a tool in the comprehensive management of the COPD patient. This “tool” has great potential to enhance the delivery and improve the efficiency of our care. Its positive attributes were underscored during the COVID-19 pandemic, when the traditional delivery of medical care was often not possible, and virtual medicine was able to provide much-needed services to patients in need. This should provide the impetus for further research into its effectiveness. The mostly mixed results in the studies described above should not be unexpected in view of patient complexity and the heterogeneity of telemedicine applications; therefore, the specifics of the technological interventions remain to be determined. Other than in relatively uncommon breakthroughs, science builds one brick at a time. The same scenario will probably play out for telemedicine in COPD.

## Data Availability

Not applicable.
